# Iroquois Homeobox Protein 2 Identified as a Potential Biomarker for Parkinson’s Disease

**DOI:** 10.3390/ijms21103455

**Published:** 2020-05-14

**Authors:** Hyuna Sim, Joo-Eun Lee, Hee Min Yoo, Sunwha Cho, Hana Lee, Aruem Baek, Jisun Kim, Hyemyung Seo, Mi-Na Kweon, Hyung Gun Kim, Young-Joo Jeon, Mi-Young Son, Janghwan Kim

**Affiliations:** 1 Stem Cell Convergence Research Center, Korea Research Institute of Bioscience and Biotechnology (KRIBB), Daejeon 34141, Korea; hyunasim@kribb.re.kr (H.S.); jooeunlee@kribb.re.kr (J.-E.L.); swcho@kribb.re.kr (S.C.); hnlee@kribb.re.kr (H.L.); areumbaek@kribb.re.kr (A.B.); jeonyj@kribb.re.kr (Y.-J.J.); 2 Department of Functional Genomics, KRIBB School of Bioscience, University of Science and Technology, Daejeon 34113, Korea; 3 Group for Biometrology, Korea Research Institute of Standards and Science (KRISS), Daejeon 34113, Korea; hmy@kriss.re.kr; 4 Department of Molecular & Life Sciences, College of Science & Technology, Hanyang University, Ansan 15588, Korea; JisunKim@hangyang.ac.kr (J.K.); hseo@hanyang.ac.kr (H.S.); 5 Mucosal Immunology Laboratory, Department of Convergence Medicine, University of Ulsan College of Medicine/Asan Medical Center, Seoul 05505, Korea; mnkweon@amc.seoul.kr; 6 Department of Pharmacology, College of Medicine, Dankook University, Cheonan 31116, Korea; hgkimm@dankook.ac.kr

**Keywords:** Parkinson’s disease, *LRRK2* G2019S, intestinal organoid, pluripotent stem cells, diagnostic marker, *IRX2*

## Abstract

The diagnosis of Parkinson’s disease (PD) is initiated after the occurrence of motor symptoms, such as resting tremors, rigidity, and bradykinesia. According to previous reports, non-motor symptoms, notably gastrointestinal dysfunction, could potentially be early biomarkers in PD patients as such symptoms occur earlier than motor symptoms. However, connecting PD to the intestine is methodologically challenging. Thus, we generated in vitro human intestinal organoids from PD patients and ex vivo mouse small intestinal organoids from aged transgenic mice. Both intestinal organoids (IOs) contained the human *LRRK2* G2019S mutation, which is the most frequent genetic cause of familial and sporadic PD. By conducting comprehensive genomic comparisons with these two types of IOs, we determined that a particular gene, namely, Iroquois homeobox protein 2 (*IRX2*), showed PD-related expression patterns not only in human pluripotent stem cell (PSC)-derived neuroectodermal spheres but also in human PSC-derived neuronal cells containing dopaminergic neurons. We expected that our approach of using various cell types presented a novel technical method for studying the effects of multi-organs in PD pathophysiology as well as for the development of diagnostic markers for PD.

## 1. Introduction

Parkinson’s disease (PD) is a common and complex neurodegenerative disorder that results from the progressive loss of midbrain dopaminergic neurons in the substantia nigra (SN). The main cause of PD may be a complicated interplay of genetic and environmental factors [1]. However, it is now recognized that the disease is a multisystem disorder involving both motor and non-motor symptoms associated with the central and peripheral nervous systems [2]. Throughout the past few decades, several genes associated with PD have been discovered through genome-wide association studies (GWAS), such as the alpha-synuclein (*SNCA*), glucocerebrosidase (*GBA*), and leucine-rich repeat kinase 2 (*LRRK2*) genes [3,4]. For the diagnosis of PD, clinical criteria based on the cardinal signs of PD and brain imaging of structural, functional, and molecular changes have been used. However, typical motor symptoms occur when up to 60% of the dopaminergic neurons of the SN have already degenerated [5]. It is, therefore, essential to develop an early diagnosis marker for the successful treatment for PD. According to previous studies, the onset of motor symptoms can be preceded by a premotor or prodromal phase characterized by specific non-motor symptoms [6]. These include emotional problems, cognitive dysfunction, sleep disturbances, sensory manipulation, as well as autonomic dysregulations [7]. Gastrointestinal (GI) dysfunction is a common non-motor symptom of PD and is observed at all stages through GI symptoms, including dysphagia, gastroparesis, and constipation [8]. The results of several studies suggest that infrequent bowel movements or constipation severity are associated with an elevated risk of PD in the future [9,10]. Moreover, the pathogenic accumulation of phosphorylated alpha-synuclein protein, the neuropathological hallmark of PD, in the bowel can occur during the early stages of the disease, prior to the onset of motor symptoms [11]. The risk of PD was lower in patients who underwent truncal vagotomy, which indicates the linkage between the brain and the GI tract is influential in the development of PD [12,13].

The pathological mutations of *LRRK2* affect protein functions and molecular pathways involved in PD [14]. In particular, Gly2019Ser substitution (G2019S) in *LRRK2*, a common genetic variation of familial and sporadic PD, increases kinase activity and induces toxicity [15]. A recent study has identified the mutated *LRRK2* gene through exome sequencing of patients with Crohn’s disease (CD) and ulcerative colitis (UC), which indicates that *LRRK2* mutations are associated with intestinal inflammatory disorders [16]. Nevertheless, the molecular association between PD-related mutations and intestinal dysfunction remains unclear. Thus, the study of gene expression profiles in PD patient-derived intestinal samples could provide clues to the pathogenesis of PD. In our previous study, in vitro intestinal organoids (IOs) and neuroectodermal spheres (NESs) derived from PD patient’s pluripotent stem cells (PSCs) showed a highly distinct gene expression patterns [17].

Here, we generated PD-relevant three-dimensional (3D) human IOs from PD patient-specific PSCs and mouse IOs from aged mouse intestines harboring the *LRRK2* G2019S mutation. Furthermore, we performed transcriptome profiles of human and mouse IOs to determine the molecular association and found a candidate gene: *IRX2*. This newly identified gene exhibited PD-related expression patterns in PD patient-derived neural cells, including NESs, and neuronal cells containing dopaminergic neurons.

## 2. Results

### 2.1. Generation of Mouse Intestinal Organoids from Human LRRK2 G2019S Transgenic Mice and Littermate Controls

In our previous study, we observed the comprehensive molecular changes caused by the *LRRK2* G2019S mutation in neural and intestinal 3D culture model systems based on the same PD-specific PSCs [17]. One of the most interesting aspects of the results was that the gene expression difference was more distinct in human intestinal organoids (hIOs) than in human neuroectodermal spheres (hNESs), even though PD is a well-known neurodegenerative disorder. To explore the potential of PD patient-derived hIOs and investigate molecular targets from *LRRK2* mutations in PD, we compared differentiated PD patient-derived IOs with ex vivo IOs from PD model mice through microarray analysis. Differentially expressed genes (DEGs) were collected to identify common factors to study the signature of PD concomitants with the intestine.

**Figure 1:**
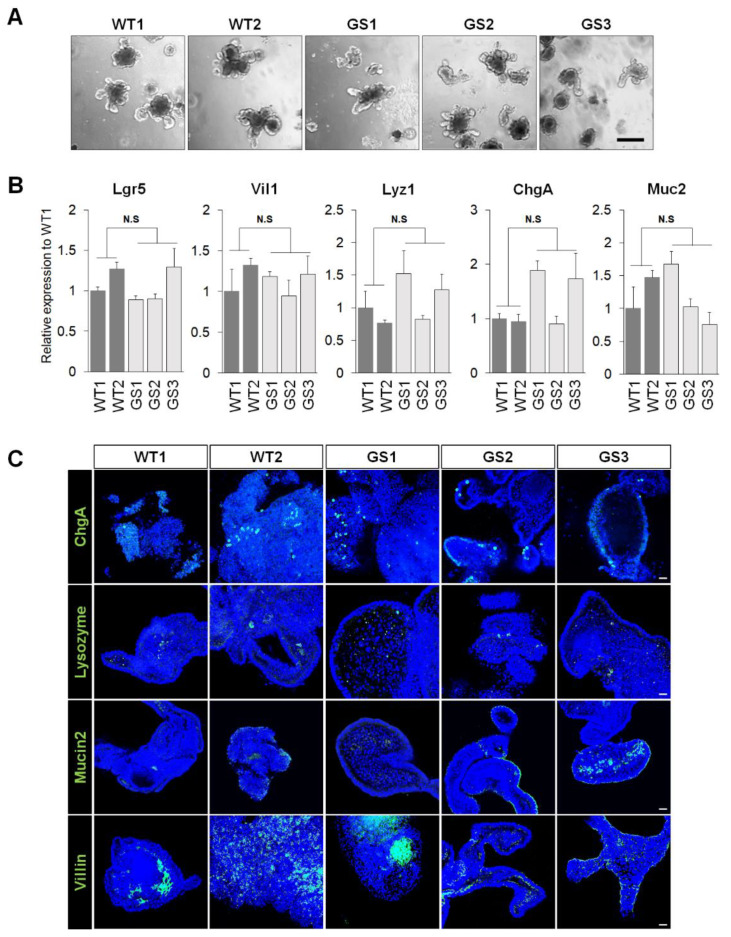
Establishment of intestinal organoids as an ex vivo model. (**A**) Isolated mouse intestinal crypts efficiently formed small intestinal organoids. (scale bar 200 μm) Wild-type (WT), Non-transgenic littermate mouse small intestinal organoids; Leucine-rich repeat kinase 2 (*LRRK2*) Gly2019Ser substitution (G2019S) (GS), The human *LRRK2* G2019S mutant mouse small intestinal organoids. (**B**) mRNA expression level of Leucine-rich repeat-containing G-protein coupled receptor 5 (*Lgr5*) (WT, 1.134 ± 0.1343, *n* = 2; GS, 1.027 ± 0.1325, *n* = 3, *p*-value 0.6272), Villin 1 (*Vil1*) (WT, 1.161 ± 0.1613, *n* = 2; GS, 1.115 ± 0.8476, *n* = 3, *p*-value 0.7929), Lysozyme 1 (*Lyz1*) (WT, 0.8842 ± 0.1158, *n* = 2; GS, 1.206 ± 0.2031, *n* = 3, *p*-value 0.3275), Chromogranin A (*ChgA*) (WT, 0.9741 ± 0.02588, *n* = 2; GS, 1.507 ± 0.3067, *n* = 3, *p*-value 0.2712), and Mucin 2 (*Muc2*) (WT, 1.235 ± 0.2354, *n* = 2; GS, 1.150 ± 0.2728, *n* = 3, *p*-value 0.8427). Data are mean ± standard error of means (SEM) of at least three independent experiments. *p*-values were analyzed using the unpaired two-tailed Student’s *t*-test (N.S, not significant). (**C**) Confocal image for ChgA (enteroendocrine cells), Lysozyme (paneth cells), Mucin2 (goblet cells), and Villin (enterocytes). Counterstain, Hoechst 33342 (scale bar 200 μm).

To reflect age-dependent accumulative PD characteristics, we generated mouse small intestinal organoids (mIOs) from human *LRRK2* G2019S transgenic (TG) mice and normal control littermates at 15–18 months of age. Ex vivo cultured mouse’s small intestinal epithelial cells exhibited a bud-like structure after 4 to 7 days of culture on Matrigel (Figure 1A). There were no differences in the forming efficiency, size, and budding structures between the human *LRRK2* G2019S mutant mouse small intestinal organoids (GS mIOs) and non-transgenic littermate mouse small intestinal organoids (WT mIOs). GS mIOs were morphologically indistinguishable from WT mIOs after several passages. Moreover, the qRT-PCR analysis showed that GS mIOs and WT mIOs contained similar expression levels of intestinal cell markers, including *Lgr5* (an intestinal stem cell marker), *Vil1* (villin 1 for enterocytes), *Lyz1* (lysozyme for Paneth cells), *ChgA* (chromogranin A for enteroendocrine cells), and *Muc2* (mucin 2 for goblet cells) (Figure 1B). We assessed the expression and localization of the intestinal cell markers via immunostaining (Figure 1C). Despite the fact that the behavioral symptoms of PD induced by the expressions of mutant *LRRK2* are found in mice of 12 months and older [18], ex vivo cultured GS mIOs showed similar characteristics to WT mIOs.

### 2.2. Comparative Transcriptome Analysis of Ex Vivo Cultured mIOs and In Vitro Differentiated hIOs

**Figure 2:**
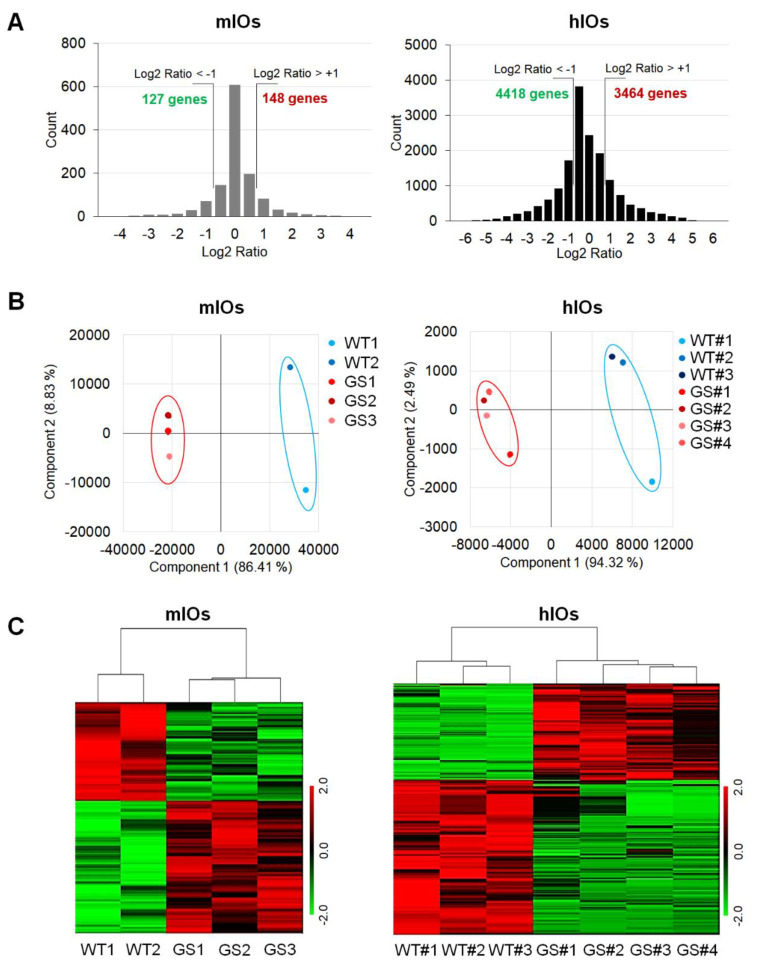
Comparative analysis of the transcriptome of ex vivo cultured mouse intestinal organoids (mIOs) and in vitro differentiated human pluripotent stem cell (PSC)-derived intestinal organoids (hIOs). (**A**) Distribution of transcriptome expression ratios in mIOs and hIOs. The Log2 ratio was calculated for each transcript. The frequencies of the Log2 ratio were plotted as a bar chart. (**B**) Principal component analysis (PCA) of the differentially expressed genes (DEGs) based on the microarray data. (**C**) Hierarchically clustered heatmap of mIOs and hIOs. Genes with expressions that were not significantly altered were removed. WT, normal control; GS, Parkinson’s disease-relevant cells, carrying *LRRK2* G2019S mutation. Both WT1 mIOs and WT#1 hIOs were used as controls.

To understand the alterations in PD using two types of PD-intestine models, we decided to perform microarray analysis to determine the genes with expression levels that were affected by the *LRRK2* G2019S mutation. First of all, we selected DEGs that were significantly altered by the *LRRK2* G2019S mutation and analyzed transcriptome alterations (Figure 2). Compared to WT mIOs, 1225 genes were significantly up- or down-regulated in GS mIOs. According to a fold-change threshold of 2.0 between WT mIOs and GS mIOs, 148 genes were up-regulated, and 127 genes were down-regulated (Figure 2A, left). In addition, we re-analyzed our previous microarray data associated with pluripotent stem cell (PSC)-derived differentiated hIOs of *LRRK2* G2019S PD patients [17] to select the common factors between the two types of models. There was a substantially greater number of significantly altered genes (16,067 genes) in hIOs with the *LRRK2* G2019S mutation compared to in healthy control PSC-derived hIOs. Accordingly, greater numbers of up-regulated (3464) and down-regulated (4418) genes were identified in hIOs with the *LRRK2* G2019S mutation when we applied a fold-change threshold of 2.0 (Figure 2A, right). The principal component analysis showed that the *LRRK2* G2019S mutant and the control were separated in both mIOs and hIOs (Figure 2B). Heatmap analysis also showed partial segregation of the expression patterns (Figure 2C). These results suggested that the gene expression profiles were altered by the *LRRK2* G2019S mutation in ex vivo cultured and PSC-derived differentiated IOs.

### 2.3. Identification and Validation of Common Factors between Two Types of Intestinal Organoids of PD

**Figure 3:**
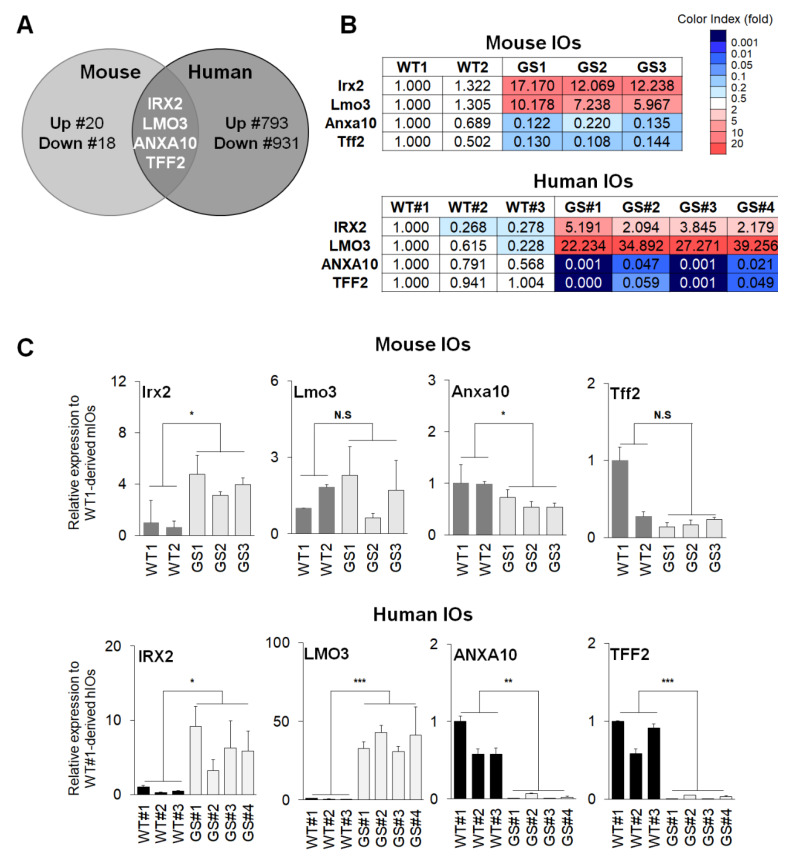
Identification and validation of commonly up-regulated or down-regulated factors based on the transcriptome analysis. (**A**) Venn diagram of the identification of commonly regulated factors between mIOs and hIOs. DEGs were selected on the basis of a >5-fold change (FC). Four factors (Iroquois homeobox 2; *IRX2*, LIM domain only 3; *LMO3*, AnnexinA10; *ANXA10,* and Trefoil factor 2; *TFF2*) were affiliated to the intersection. (**B**) Transcriptome analysis data of commonly regulated genes. (**C**) Quantitative Real-Time PCR (qRT-PCR) verification of commonly regulated genes, including *Irx2* (WT, 0.8132 ± 0.1868, *n* = 2; GS, 3.953 ± 0.4730, *n* = 3, *p*-value 0.0153), *Lmo3* (WT, 1.414 ± 0.4138, *n* = 2; GS, 1.526 ± 0.4898, *n* = 3, *p*-value 0.8830), *Anxa10* (WT, 0.9925 ± 0.007453, *n* = 2; GS, 0.5974 ± 0.06272, *n* = 3, *p*-value 0.0165), and *Tff2* (WT, 0.6377 ± 0.3623, *n* = 2; GS, 0.1783 ± 0.02823, *n* = 3, *p*-value 0.1903) in mIOs and *IRX2* (WT, 0.5759 ± 0.2178, *n* = 3; GS, 6.148 ± 1.224, *n* = 4, *p*-value 0.0124), *LMO3* (WT, 0.5873 ± 0.2296, *n* = 3; GS, 36.96 ± 2.965, *n* = 4, *p*-value 0.0001), *ANXA10* (WT, 0.7185 ± 0.1408, *n* = 3; GS, 0.02325 ± 0.01537, *n* = 4, *p*-value 0.0021). and *TFF2* (WT, 0.8333 ± 0.1256, *n* = 3; GS, 0.02061 ± 0.01213, *n* = 4, *p*-value 0.0006) in hIOs. FCs in the expression levels were relative to WT1 mIOs and WT#1 hIOs, respectively. Data are mean ± standard error of means (SEM) of at least three independent experiments. *p*-values were analyzed using the unpaired two-tailed Student’s *t*-test (* *p* < 0.05, ** *p* < 0.005, *** *p* < 0.001, N.S, not significant).

Through transcriptome analysis on the two types of PD-intestine models, we attempted to select commonly detectable up-regulated or down-regulated genes with a fold-change threshold of 5.0 between the *LRRK2* G2019S mutant and the control (Figure S1). We identified four common factors, including *IRX2* (Iroquois homeobox protein 2), *LMO3* (LIM domain only protein 3), *ANXA10* (AnnexinA10), and *TFF2* (Trefoil factor 2) (Figure 3A). Microarray data indicated that *IRX2* and *LMO3* were up-regulated in the *LRRK2* G2019S mutant, whereas *ANXA10* and *TFF2* were down-regulated (Figure 3B). The qRT-PCR analysis confirmed that the expression levels of selected genes were altered in mIOs and hIOs with similar patterns that are represented by the microarray analysis (Figure 3C).

### 2.4. Confirmation of Commonly Regulated Factors in NESs and Neuronal Cells Containing Dopaminergic Neurons

**Figure 4:**
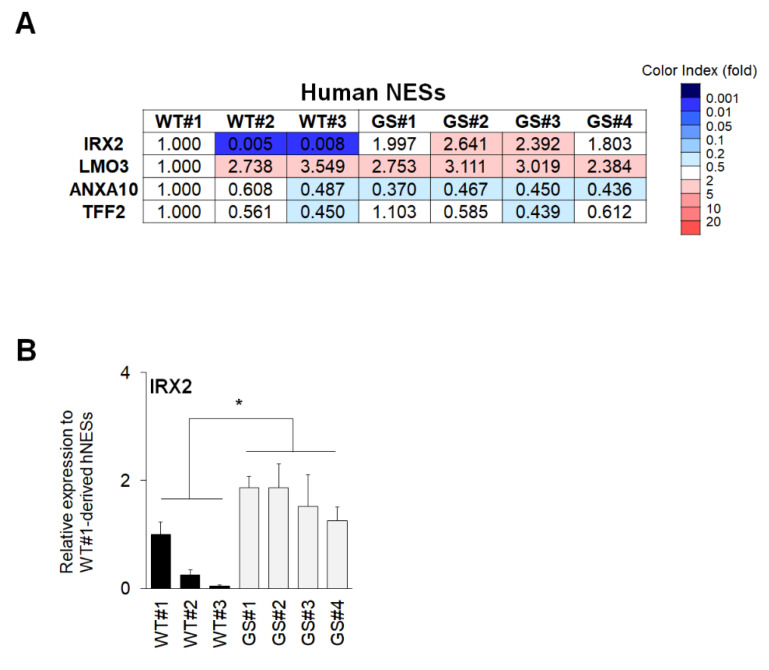
Verification of intestine-based four common factors in human neuroectodermal spheres (NESs). (**A**) Microarray data of commonly regulated genes in human NESs from a previous study. (**B**) qRT-PCR confirmation of *IRX2* (WT, 0.4329 ± 0.2898, *n* = 3; GS, 1.628 ± 0.1466, *n* = 4, *p*-value 0.0103) expression in human NESs. Data are mean ± standard error of means (SEM) of at least three independent experiments. *p*-values were analyzed using the unpaired two-tailed Student’s *t*-test (* *p* < 0.05).

Previous studies suggest that the gut-brain connection is associated with the initiation and progression of PD [19]. Therefore, we investigated the common factors of intestinal organoids on neural cells. First, we checked the expression patterns of *IRX2, LMO3, ANXA10,* and *TFF2* in human PSC-derived NESs (Figure 4A) [17]. Only *IRX2* showed a similar pattern, which indicated that the *IRX2* expression was significantly up-regulated in human *LRRK2* G2019S PD patient-derived NESs compared to in wild-type control NESs. We also validated the expression levels through qRT-PCR analysis (Figure 4B).

**Figure 5:**
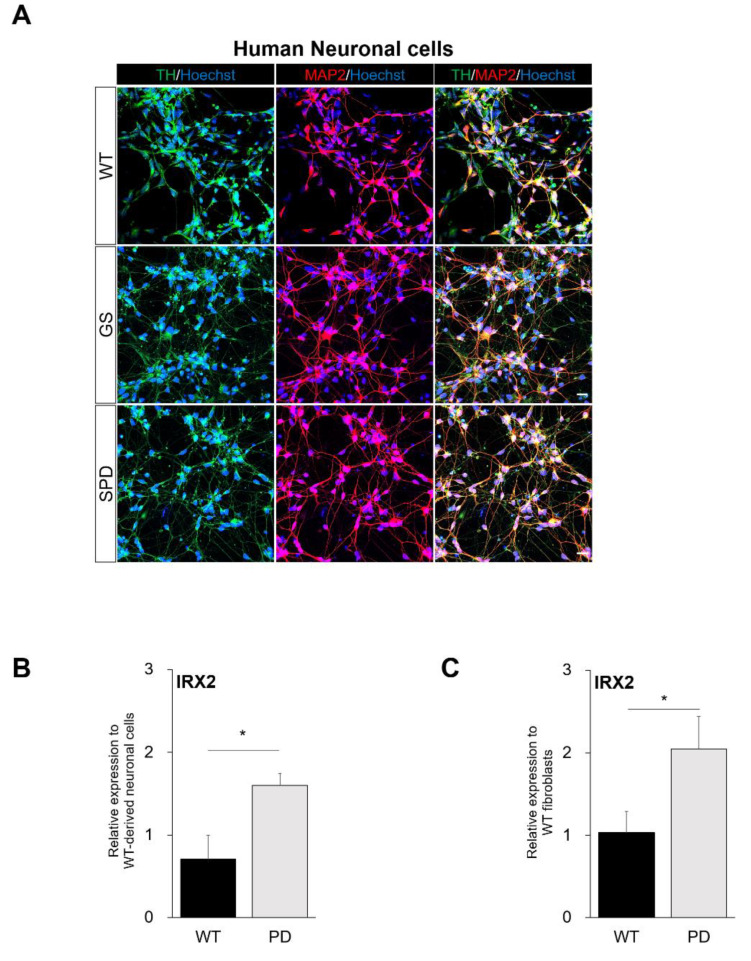
Confirmation of the expression of the newly identified gene, *IRX2*, in neuronal cells containing dopaminergic neurons. (**A**) Characterization of neuronal cells containing dopaminergic neurons from healthy control (WT), *LRRK2* G2019S familial (GS), and sporadic (SPD) PSCs. The neuronal marker (Microtubule-associated protein 2; MAP2) and the dopaminergic neuronal marker (Tyrosine hydroxylase; TH) were co-stained (scale bar 20 μm). (**B**) Relative expression levels of *IRX2* (WT, 0.7100 ± 0.2900, *n* = 2; PD, 1.598 ± 0.1473, *n* = 5, *p*-value 0.0281) in familial/sporadic PD patient-derived neuronal cells containing dopaminergic neurons (PD) in comparison with normal control-derived cells (WT). (**C**) Determination of *IRX2* in human fibroblast cells. Examination of *IRX2* (WT, 1.033 ± 0.2582, *n* = 8; PD, 2.047 ± 0.3931, *n* = 8, *p*-value 0.0489) expression in familial/sporadic PD patient-derived fibroblast (PD) and healthy control fibroblast (WT) through qRT-PCR analysis. Data are mean ± standard error of means (SEM) of at least three independent experiments. *p*-values were analyzed using the unpaired two-tailed Student’s t-test (* *p* < 0.05).

According to previous reports, familial PD patients harboring the G2019S mutation in the *LRRK2* gene were clinically indistinguishable from idiopathic PD, showing similarities in terms of motor deficits [20], clinical features [21,22], and neurochemical phenotypes [23]. Thus, we differentiated human PSCs from familial PD patients harboring the *LRRK2* G2019S mutation and from sporadic PD patients into neuronal cells containing dopaminergic neurons, which are known to be affected by PD progression. Thirty-five days differentiated neuronal cells exhibited the expression of the dopaminergic neuron-specific marker tyrosine hydroxylase (TH) and the mature neuron marker microtubule-associated protein 2 (MAP2) (Figure 5A). We confirmed cell survival and analyzed TH and MAP2 double-positive neuronal cells from familial/sporadic PD and healthy control (WT)-derived induced pluripotent stem cells (Figure S2). We found that there were no differences in neuronal cell death between WT and PD through the expression levels of cleaved caspase-3 (Figure S2A,B). In addition, there were no significant differences between healthy control (WT) and the PD-group in terms of the expressions of TH and MAP2 double-positive cells (Figure S2C). We detected elevated expressions of *IRX2* in familial *LRRK2* G2019S and sporadic PD patient-derived neuronal cells in comparison with wild-type control cells (Figure 5B), as shown in human *LRRK2* G2019S PD patient-derived IOs and NESs. Finally, we also checked the expression levels of *IRX2* in familial *LRRK2* G2019S and sporadic PD patient-derived fibroblasts to determine the feasibility of *IRX2* as a potential biomarker (Figure 5C). Interestingly, *IRX2* showed similar expression patterns in both familial and sporadic PD patient-derived fibroblasts, which are known as appropriate samples for diagnostic purposes. In summary, these data suggested that significantly increased expression levels of *IRX2* could be used as a PD-specific biomarker.

## 3. Discussion

Human development and disease are challenging topics of study due to the low experimental availability of in vivo systems and the complex biological networks that are involved [24]. Although experimental animal models have been used to study the pathogenesis of the disease, the use of model organisms often does not translate into the replication of human biology as such models possess different structures and physiology [25,26]. For these reasons, 3D organoid culture systems are more preferred in recent years and serve as an excellent platform for modeling human tissue or organs.

Organoids have been widely adopted in disease pathology, regenerative medicine, and drug development, including in pharmacokinetics analysis, efficacy evaluation, and toxicity testing [27]. In particular, 3D organotypic cultures from disease-specific PSCs represent a greater number of relevant characteristics and symptoms of complex human diseases [28,29]. Accordingly, patient-specific 3D organoid models represent a powerful tool for investigating disease pathology.

In our previous study, we observed the comprehensive molecular changes caused by the *LRRK2* G2019S mutation in 3D culture model systems based on PD PSCs. The expression profiles of PD patient-derived cells were distinct from those of wild-type controls in hIOs as well as hNESs containing neural progenitor cells. This patient-derived 3D culture model system resulted in extensive alterations in global gene expression, such as synaptic transmission, specifically synaptic vesicle trafficking, and defects of which are known to be related to PD [17]. As a result, IOs harboring the *LRRK2* G2019S mutation could serve as a versatile model to explain the association between PD and intestinal alterations.

A number of studies have shown that *LRRK2* affects pathophysiological modifications in the GI pathogenesis of PD [6,7,10,17]. The mechanism of *LRRK2*-associated GI pathogenesis has been examined in *LRRK2* TG mice and knockout mice [30,31,32]. Thus, in this study, we developed mIOs as an ex vivo system and compared them with differentiated hIOs through transcriptome analysis to identify molecular targets that are regulated by *LRRK2* mutation. The results showed that the two types of IOs exhibited distinctive gene expressions induced by the *LRRK2* G2019S mutation. We subsequently selected the genes that were significantly up-regulated or down-regulated in the two types of intestinal organoids harboring *LRRK2* G2019S to identify the common factors. As a result, four genes were selected: *IRX2, LMO3, ANXA10,* and *TFF2*. Two genes (*IRX2, LMO3*) were up-regulated, and the remaining two (*ANXA10, TFF2*) were down-regulated by the expression of mutant *LRRK2*. Taken together, our results suggested that these genes might be potential factors that could possibly emerge in PD pathology.

According to a recent report, the gut-to-brain propagation of pathogenic proteins induces PD through dopaminergic neurodegeneration [33]. Thus, it is worthwhile to verify the expression levels of the selected genes from IOs models of PD in neural cells. Notably, *IRX2*, one of the selected genes from the *LRRK2* G2019S IOs models, exhibited a similar expression pattern in hNESs from *LRRK2* G2019S PD patient-derived PSCs. *IRX2* is a member of the Iroquois homeobox genes that are expressed in the cerebellum and applies multiple functions during animal development [34,35]. Vertebrate *Irx* genes participate in the regionalization of the brain and have been reported to exhibit functions in neural development [35]. On the other hand, *Irx2*-deficient mice have shown normal developmental phenotypes through the dynamic expression of Irx2 in the developing heart, nervous system, and other organs [36]. Most vertebrates include six genes and two clusters of three genes, with the IrxA cluster comprised of *Irx1, Irx2,* and *Irx4,* and the IrxB cluster comprised of *Irx3, Irx5,* and *Irx6* [37,38]. Recently, GWAS of PD has discovered the potential functionality of risk correlated with single nucleotide polymorphisms (SNPs) based on non-coding DNA, including *IRX2, 3,* and *6* [39]. An additional transcriptome mapper (TRAM) profiling has been conducted in post–mortem whole SN tissue and single dopamine neurons from familial and sporadic PD patients to identify chromosomal segments and gene loci diverse expression. TRAM has determined 10 significant genes, including *IRX2*, that have been up-regulated or down-regulated in SN and midbrain dopaminergic neurons of PD patients [40]. In addition, mass spectrometry-based phosphoproteomics of genetically engineered *LRRK2* mice has led to the discovery of several key substrates, including the *Irx1* gene [41]. Accordingly, we were able to confirm the elevated expressions of *IRX2,* which was also discovered in *LRRK2* G2019S PD patient PSC-derived neuronal cells containing dopaminergic neurons. Even though previous studies have shown conflicting results [34,35,36], the evolutionarily conserved *IRX2* gene, which showed elevated expression patterns not only in neural cells but also in IOs, would conceivably be associated with PD progression. In addition, we also investigated the expression patterns of the Iroquois family of homeobox genes in hNESs and IOs (Figure S3). In hIOs, six genes were up-regulated in the *LRRK2* G2019S PD patient-derived cells when compared to the wild-type control cells. However, the IrxA cluster (*IRX1*, *IRX2*, and *IRX4*) showed a significantly greater increase in the *LRRK2* G2019S PD patient-derived cells over the wild-type control cells compared to the IrxB cluster (*IRX3*, *IRX5*, and *IRX6*) in hNESs. We further examined the expression of *IRX2* in fibroblasts of familial and sporadic PD patients. *IRX2* showed an expression pattern similar to those shown in the intestinal and neural models of PD. Through these results, we suggested that increased levels of *IRX2* could be used to detect PD in various cell types, including fibroblasts as well as intestinal and neuronal cells containing dopaminergic neurons. Although we were not able to determine the role of the increase in IRX2 in neural and intestinal cells during the progression of PD and underline the mechanisms in this study, the newly discovered IRX2 is expected to be an interesting factor for upcoming research. It may be worthwhile to check the expression levels of IRX2 with a larger group of PD patients.

All in all, *IRX2* could be a promising factor in studying molecular alterations in PD and could be used as a diagnostic marker for PD. Furthermore, intestinal organoids could be appropriate PD models even though they are composed of the only epithelium. Our multi-organ culture system could lead to a better understanding of PD.

## 4. Materials and Methods

### 4.1. Animals

*LRRK2* transgenic mice expressing the G2019S mutation [FVB/N-Tg (LRRK2*G2019S) 1Cjli/J] were purchased from Jackson Laboratory (Bar Harbor, ME, USA). The mice were maintained under a 12 h light/dark cycle and given free access to food and water. All experimental procedures followed guidelines approved by the Institutional Animal Care and Use Committee of Hanyang University (HY-IACUC-12-018).

### 4.2. Generation of Mouse Small Intestinal Organoids

Mouse small intestinal organoids (mIOs) were isolated and established according to the manufacturer’s instructions using the IntestiCult^TM^ Organoid Growth Medium (STEMCELL Technologies, Vancouver, BC, Canada). Mouse small intestines were harvested about 10–20 cm in length. Fine forceps were used to remove the membrane, blood vessels, and fat from the exterior of the tissue. The intestinal segments were subsequently flushed with cold Dulbecco’s Phosphate-Buffered Saline (DPBS, Welgene, Gyeongsan-si, Korea) and were opened using small scissors. The intestinal sheets were thoroughly rinsed with clean DPBS, and the washed intestine sheets were chopped into 2 mm pieces. Tissue pieces were pipetted up and down gently in DPBS using a pre-wetted 10 mL serological pipette. For the dissociation of tissue pieces and isolation of crypts, the tissue pieces were treated with Gentle Cell Dissociation Reagent (STEMCELL Technologies, Vancouver, BC, Canada) for 15–20 min at room temperature. The pellets were resuspended in cold PBS containing 0.1% BSA (Sigma-Aldrich, St. Louis, MO, USA) and were subsequently filtered through a 70 μm strainer (Corning Inc., Corning, NY, USA) to obtain crypts of appropriate sizes. The selected intestinal crypts were mixed with cold DMEM/F-12 (Thermo Fisher Scientific, Waltham, MA, USA). Through counting, 3000 crypts were collected. To produce the solid dome, the crypts were resuspended in the IntestiCult^TM^ Organoid Growth Medium and mixed with Matrigel Matrix (BD Biosciences, San Jose, CA, USA) with a 1:1 ratio. The 50 µL of the crypts and Matrigel matrix mixture was incubated at 37 °C for 10 min. IntestiCult^TM^ Organoid Growth Medium was added to the sidewall of the well. The culture medium was half-exchanged every three days. Organoids were passaged using the Gentle Cell Dissociation Reagent after 10 to 14 days of culture.

### 4.3. Quantitative Real-Time PCR (qRT-PCR)

Total RNA was extracted from the organoids using the RNeasy Plus Mini Kit (QIAGEN, Hilden, Germany) and reverse-transcribed using the iScript^TM^ cDNA Synthesis Kit (Bio-Rad Laboratories, Inc., Hercules, CA, USA). The qRT-PCR was performed using the 7500 Fast Real-Time PCR System (Applied Biosystems, Foster City, CA, USA). The primers used in this study are listed in Table S1. All experiments were performed in triplicate.

### 4.4. Western Blot Analysis

Cells were treated with cold sample lysis buffer (1% Triton X-100, Xpert Protease Inhibitor Cocktail Solution (GenDEPOT, Katy, TX, USA), 5 mM Ethylenediaminetetraacetic acid (EDTA, Thermo Fisher Scientific, Waltham, MA, USA), and 1 mM Phenylmethanesulfonyl fluoride (PMSF, Thermo Fisher Scientific, Waltham, MA, USA)). Protein Assay Dye Reagent Concentrate (Bio-Rad Laboratories Inc., Hercules, CA, USA) was used for determining the protein concentration. An equal amount of protein was loaded for SDS-PAGE and transferred onto PVDF membrane using Wet/Tank Blotting Systems (Bio-Rad Laboratories Inc., Hercules, CA, USA). The membranes were incubated with 5% Difco^TM^ Skim Milk (BD Biosciences, San Jose, CA, USA) for 30 min at room temperature. The membranes were incubated with cleaved caspase-3 (Asp175) antibody (Cat. 9661S, Cell Signaling Technology, Danvers, MA, USA) and GAPDH antibody (Cat. sc-47724, Santacruz Biotechnology Inc., Dallas, TX, USA) overnight at 4 °C, followed by incubation with horseradish peroxidase (HRP)-conjugated secondary antibodies (Cell Signaling Technology, Danvers, MA, USA). We added ECL^TM^ Select Western Blotting Detection Reagent (GE Healthcare, Chicago, IL, USA) on the membrane for the detection of the signals from HRP. The images of protein bands were obtained by a LAS-3000 imaging system (Fujifilm, Minato, Tokyo, Japan).

### 4.5. Immunofluorescence of Mouse Small Intestinal Organoids and Human Neuronal Cells Containing Dopaminergic Neurons

Whole mIOs were fixed overnight in fresh 4% paraformaldehyde. Fixed organoids were unmasked in PBS through shaking at room temperature and were blocked and permeabilized with 5% BSA and 0.1% Triton X-100 in PBS. The primary antibodies that were used were Villin (Cat. sc-58897, Santa Cruz Biotechnology Inc., Dallas, TX, USA), Mucin 2 (Cat. sc-15334, Santa Cruz Biotechnology Inc., Dallas, TX, USA), Chromogranin A (Cat. ab15160, Abcam, Cambridge, UK), and Lysozyme (Cat. ab108508, Abcam, Cambridge, UK). Human neuronal cells containing dopaminergic neurons were fixed with 80% Methanol for 10 min. After blocking with 5% BSA, the primary antibodies that were used were MAP2 (Cat. ab5392, Abcam, Cambridge, UK) and TH (Cat. Ab152, Millipore Sigma, Burlington, MA, USA). The primary antibodies were bound overnight at 4 °C. The secondary antibodies that were used were donkey anti-mouse and anti-rabbit Alexa Fluor 488 and 594 (Invitrogen, Carlsbad, CA, USA). The secondary antibodies were bound for 1 h at room temperature. DNA was stained with Hoechst 33342 (Cat. H3570, Thermo Fisher Scientific, Waltham, MA, USA). The images of the crypt organoids and human neuronal cells containing dopaminergic neurons were acquired using an LSM 800 confocal microscope with Zeiss software.

### 4.6. Microarray and Transcriptome Analysis

Microarray analysis was performed using the Low Input Quick Amp Labeling Kit (Agilent Technologies, Santa Clara, CA, USA), RNase Mini-Column (QIAGEN, Hilden, Germany), and Mouse V2 Gene expression 4 × 44K (Agilent Technologies, Santa Clara, CA, USA), according to the manufacturers’ protocols. Data quantification was achieved by using the Feature Extraction software 10.7, and global normalization was conducted using GeneSpringGX 7.3.1 (Agilent Technologies, Santa Clara, CA, USA). Heatmap clustering was visualized using the MeV 4.9.0 software. Principal component analysis (PCA) of the differentially expressed genes was reflected using scikit-learn, Python package. Functional annotation clustering and the Kyoto Encyclopedia of Genes and Genomes (KEGG) pathway were analyzed using David Bioinformatics Resources 6.7 (https://david-d.ncifcrf.gov). We used relative values to compare the expression levels of the selected genes, meaning that the global normalized value of WT1 was used as the standard point of the mouse results, whereas WT#1 was used as the standard point of the human results.

### 4.7. Differentiation of Human-Induced Pluripotent Cells into Human Intestinal Organoids, Human Neuroectodermal Sphere, and Culture

Human wild-type (WT) and Parkinson’s disease patient (PD) somatic cells (AG02261, GM01680, GM01706, GM02036, GM02623, GM23967, ND14317, ND29492, ND33879, ND38262, AG20439, AG20442, AG20443, AG20445, and AG20446) were purchased from the Coriell Institute (Camden, NJ, USA). The human embryonic stem cell line (H9) was purchased from the WiCell Research Institute (Madison, WI, USA). Healthy fibroblasts (IMR90, MRC5, and CRL-2097) were purchased from the American Type Culture Collection (Manassas, VA, USA). Based on these cell lines, human intestinal organoids (hIOs) and human neuroectodermal spheres (hNESs) were generated, as previously described [17]. To differentiate hindgut spheroids, human pluripotent stem cells (PSCs) were treated with 100 ng/mL Activin A (Research and Diagnostic Systems, Inc., Minneapolis, MN, USA), 500 ng/mL Fibroblast growth factor 4 (FGF4, Research and Diagnostic Systems, Inc., Minneapolis, MN, USA), and 500 ng/mL Wnt Family Member 3A (WNT3A, Research and Diagnostic Systems, Inc., Minneapolis, MN, USA). The spheroids were cultured in an hIO media containing 1× B27 (Invitrogen, Carlsbad, CA, USA), 100 ng/mL Epidermal Growth Factor (EGF, Research and Diagnostic Systems, Inc., Minneapolis, MN, USA), 500 ng/mL R-Spondin 1 (Research and Diagnostic Systems, Inc., Minneapolis, MN, USA), and 100 ng/mL Noggin (Research and Diagnostic Systems, Inc., Minneapolis, MN, USA). To generate hNESs, Embryonic bodies (EBs) were cultured in a neural progenitor culture medium that includes DMEM/F12, 1X N2/B27 (Invitrogen, Carlsbad, CA, USA), 20 ng/mL basic Fibroblast Growth Factor (bFGF, Research and Diagnostic Systems, Inc., Minneapolis, MN, USA), 20 ng/mL EGF (PeproTech, Inc., Rocky Hill, NJ, USA), and 10 ng/mL human Leukemia inhibitory factor (LIF, PeproTech, Inc., Rocky Hill, NJ, USA). Our reference samples were purchased from the Coriell Institute (Camden, NJ, USA), WiCell Research Institute (Madison, WI, USA), and American Type Culture Collection (Manassas, VA, USA) and have been determined to be exempt from IRB review.

### 4.8. Generation of Human Neuronal Cells Containing Dopaminergic Neurons

Human neuronal cells containing dopaminergic neurons were differentiated from human PSCs, as previously described [42,43], with some modifications. At first, human PSCs were differentiated into human primitive neural stem cells (hpNSCs) with 10 ng/mL human LIF (PeproTech, Inc., Rocky Hill, NJ, USA), 4 μM CHIR99021 (Tocris Bioscience, Bristol, UK), 3 μM SB431542 (Tocris Bioscience, Bristol, UK), and 0.1 μM Compound E (Millipore Sigma, Burlington, MA, USA) for 7 days. Treatment with 2 μM Dorsomorphin (Sigma-Aldrich, St. Louis, MO, USA) was performed during the first 2 days of differentiation. The cells were subsequently passaged and maintained in 10 ng/mL human LIF, 3 μM CHIR99021, and 2 μM SB431542. The hpNSCs were treated with 100 ng/mL Sonic hedgehog (SHH, Research and Diagnostic Systems, Inc., Minneapolis, MN, USA), 100 ng/mL Fibroblast Growth Factor 8 (FGF8, Research and Diagnostic Systems, Inc., Minneapolis, MN, USA), and 2 μM Purmorphamine (Tocris Bioscience, Bristol, UK) for 7 days. Treatment with 20 ng/mL Brain-derived Neurotrophic Factor (BDNF, Research and Diagnostic Systems, Inc., Minneapolis, MN, USA), Glial cell-derived Neurotrophic Factor (GDNF, Research and Diagnostic Systems, Inc., Minneapolis, MN, USA), and 1 ng/mL Transforming Growth Factor beta-3 (TGF-b3, PeproTech, Inc., Rocky Hill, NJ, USA) was performed for 20 days to differentiate into neuronal cells containing dopaminergic neurons.

### 4.9. Statistical Analysis

Statistical analysis was performed on GraphPad Prism V5 (San Diego, CA, USA). All data are presented as means ± standard error of means (SEM). The statistical results were analyzed through the unpaired, two-tailed Student’s *t*-test. *p*-values less than 0.05 were considered as statistically significant. All assays were performed with three-five technical replicates. The number of independent experiments and standard error for each experiment are represented in the relevant manuscript and figure legends.

## 5. Conclusions

We proposed several genes as potential therapeutic targets from 3D organoid culture systems based on PD-specific mIOs and hIOs containing the *LRRK2* G2019S mutation. We observed the molecular changes caused by the *LRRK2* G2019S mutation and identified the *IRX2* gene. *IRX2* showed similar expression patterns in PSC-derived hNES and PSC-derived human neuronal cells containing dopaminergic neurons. Moreover, we could also detect the elevated expression of *IRX2* in sporadic PD patient-derived neural cells. Based on our results, we expected that the newly identified genes from our comparative analysis could be used in studying PD pathology and developing diagnostic markers for familial and sporadic PD.

## Supplementary Materials

The following are available online at https://www.mdpi.com/1422-0067/21/10/3455/s1, Figure S1. Hierarchically clustered heatmap of the differentially expressed genes between the *LRRK2* G2019S mutant and the control with a fold-change threshold of 5.0 in (A) mIOs and (B) hIOs. Figure S2. Analysis of WT and PD-derived neuronal cells containing dopaminergic neurons. Figure S3. Expression patterns of the Iroquois family of homeobox genes in hIOs and hNESs. Table S1. Primer sequences used in this study.

## Abbreviations

3D	three-dimensional
ANXA10	AnnexinA10
CD	Crohn’s disease
DEGs	Differentially expressed genes
G2019S	Gly2019Ser substitution
GBA	Glucocerebrosidase
GI	Gastrointestinal
GS mIOs	Human *LRRK2* G2019S mutant mouse small intestinal organoids
GWAS	Genome-wide association studies
hIOs	Human intestinal organoids
hNESs	Human neuroectodermal spheres
IOs	Intestinal organoids
iPSCs	Induced pluripotent stem cells
IRX2	Iroquois homeobox protein 2
LMO3	LIM domain only protein 3
LRRK2	Leucine-rich repeat kinase 2
MAP2	Microtubule-associated protein 2
mIOs	Mouse small intestinal organoids
NESs	Neuroectodermal spheres
PD	Parkinson’s disease
PSC	Pluripotent stem cells
SN	Substantia nigra
SNCA	Alpha-synuclein
SNPs	Single nucleotide polymorphisms
TFF2	Trefoil factor 2
TG	Transgenic
TH	Tyrosine hydroxylase
TRAM	Transcriptome Mapper
UC	Ulcerative colitis
WT mIOs	Non-transgenic littermate mouse small intestinal organoids
