# Supplementary figures and images for: Iroquois Homeobox Protein 2 Identified as a Potential Biomarker for Parkinson’s Disease
Source: Int J Mol Sci. 2020 May 14;21(10):3455. doi: 10.3390/ijms21103455 (PMC7278941; doi:10.3390/ijms21103455)

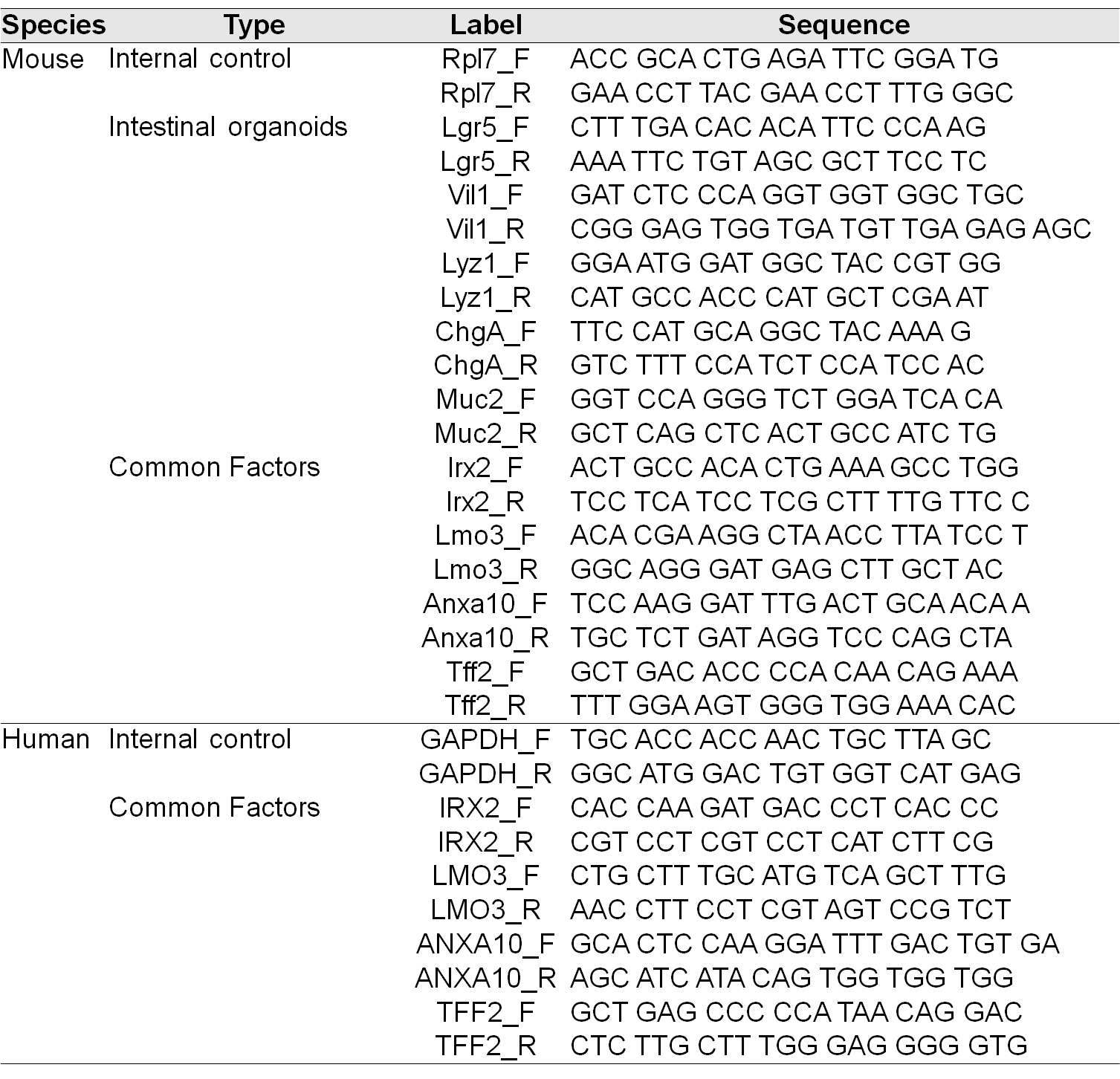

Supplement: Supplementary file 1 [file ijms-21-03455-s001.zip › Table 1.tif]

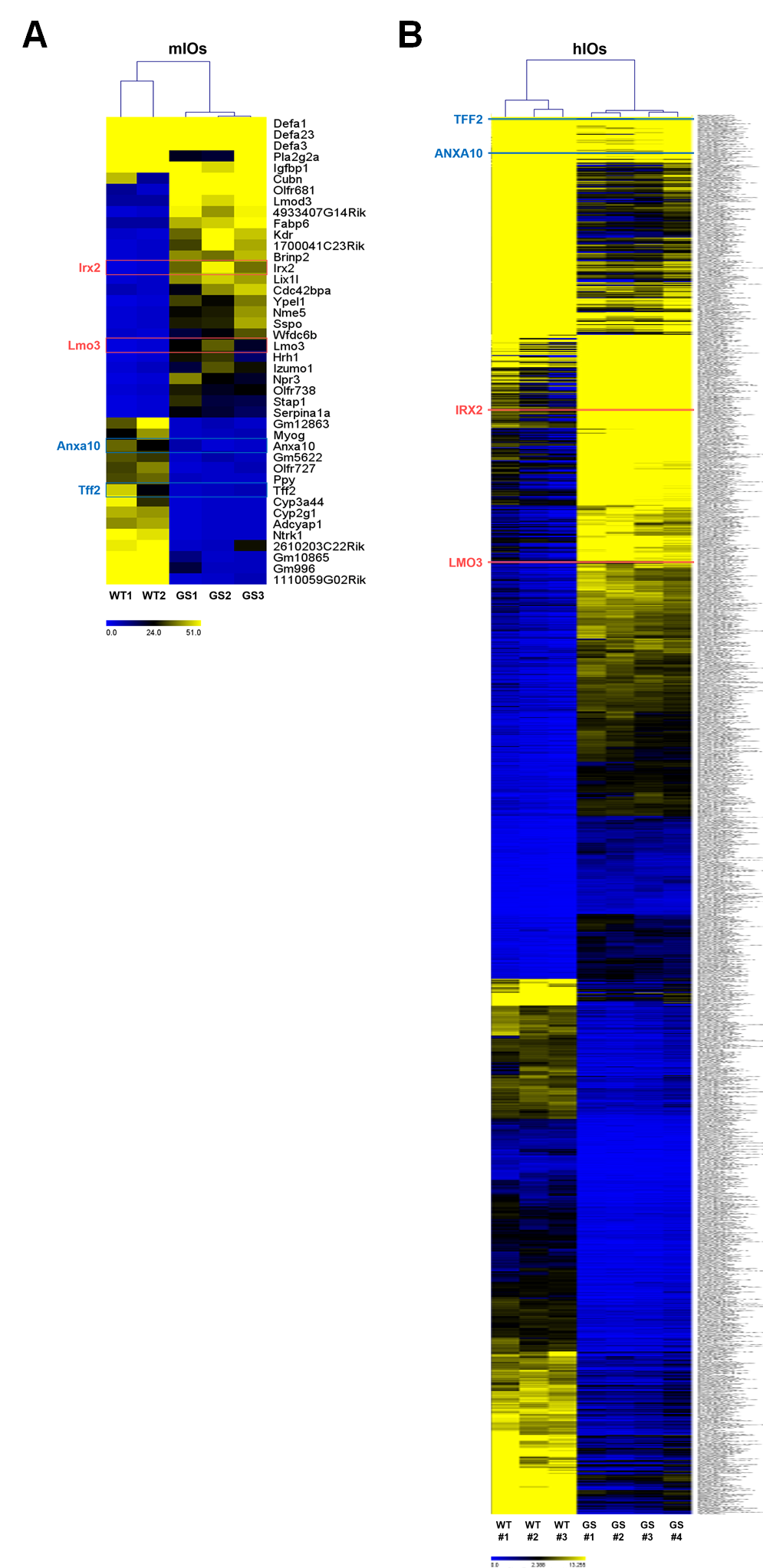

Supplement: Supplementary file 1 [file ijms-21-03455-s001.zip › Figure S1.tif]

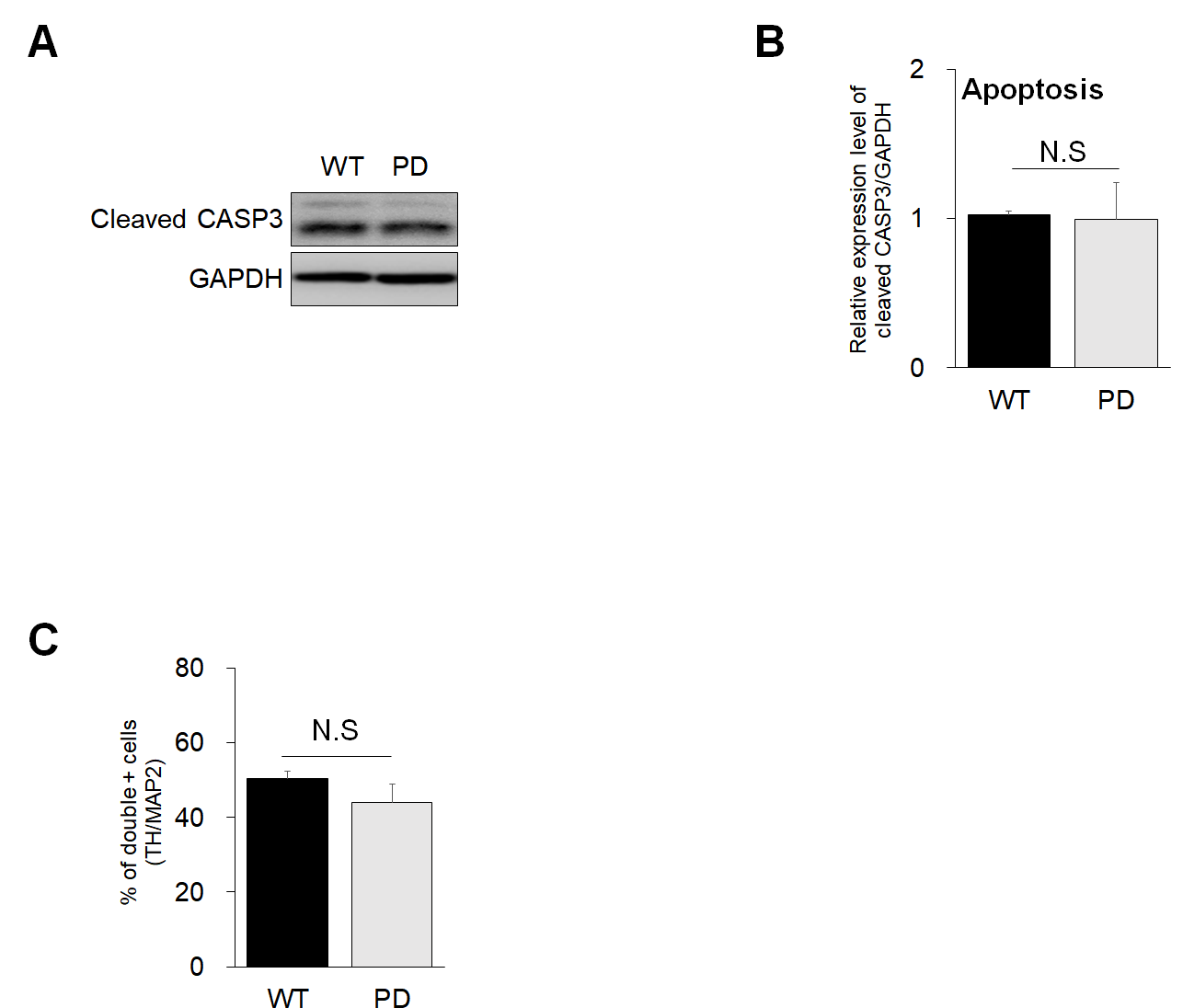

Supplement: Supplementary file 1 [file ijms-21-03455-s001.zip › Figure S2.tif]

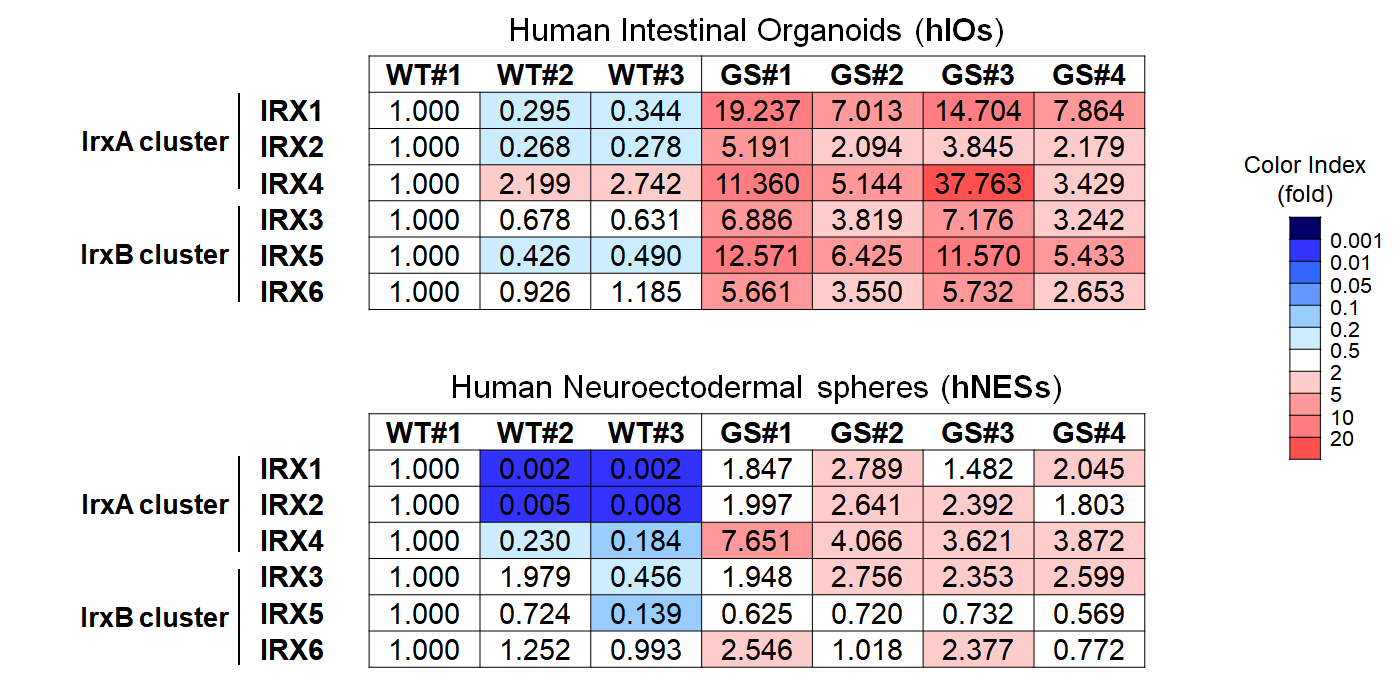

Supplement: Supplementary file 1 [file ijms-21-03455-s001.zip › Figure S3.tif]
